# Low prevalence of HTLV1/2 infection in a population of immigrants living in southern Italy

**DOI:** 10.1371/journal.pntd.0006601

**Published:** 2018-06-25

**Authors:** Loredana Alessio, Carmine Minichini, Mario Starace, Laura Occhiello, Mara Caroprese, Giovanni Di Caprio, Caterina Sagnelli, Luciano Gualdieri, Mariantonietta Pisaturo, Lorenzo Onorato, Gaetano Scotto, Margherita Macera, Stefania De Pascalis, Evangelista Sagnelli, Nicola Coppola

**Affiliations:** 1 Infectious Diseases Unit, AORN Sant’ Anna and San Sebastiano, Caserta, Italy; 2 Medical Center, Centro Sociale ex Canapificio, Caserta, Italy; 3 Medical Center, Centro di Accoglienza “La tenda di Abramo”, Caserta, Italy; 4 Department of Mental Health and Public Medicine, Section of Infectious Diseases, University of Campania Luigi Vanvitelli, Naples, Italy; 5 Medical center, Centro Suore Missionarie della Carità, Naples, Italy; 6 Medical Center, Centro per la Tutela della Salute degli Immigrati, Naples, Italy; 7 Infectious Diseases Unit, Foggia, Italy; George Washington University, UNITED STATES

## Abstract

**Aims:**

To assess the prevalence of HTLV-1 and HTLV-2 infections in a cohort of immigrants living in southern Italy.

**Findings:**

We screened for antibody to HTLV-1/2 infection 1,498 consecutive immigrants born in endemic areas (sub-Saharan Africa or southern-Asia) by a commercial chemiluminescent microparticle immunoassay. If confirmed in a Western blot assay, which differentiates anti-HTLV-1 from anti-HTLV-2, the positive sera were tested for specific HTLV RNA by a home-made PCR. The immigrants investigated were more frequently males (89.05%), young (median age 26 years), with a low level of education (median schooling 6 years), born in sub-Saharan Africa (79.70%). They had been living in Italy for a median period of 5 months. Only one (0.07%) subject was anti-HTLV-1 -positive/HTLV-1 RNA-negative; he was an asymptomatic 27-year-old male from Nigeria with 6 years’ schooling who stated unsafe sexual habits and unsafe injection therapy.

**Conclusions:**

The data suggest screening for HTLV1 and HTLV-2 infections all blood donors to Italy from endemic countries at least on their first donation; however, a cost-effectiveness study is needed to clarify this topic.

## Author summary

As in Italy the immigrants constitute one tenth of the whole population and its contribution to blood donation is strongly desired we believe that to start screening for HTLV-1 and HTLV-2 infections in all donors from endemic countries at least on their first donation is a practice useful for a correct cost-effectiveness analysis and for a conclusive decision on this topic.

## Introduction

Human T cell leukemia virus type 1 (HTLV-1) or 2 (HTLV-2) infection has a worldwide distribution, with an estimate of up to 15–20 million people affected [1]. The prevalence changes substantially according to the geographical area, and is higher in specific risk groups such as intravenous drug users and sex workers [2,3]. Endemic areas for HTLV-1 infection have been reported in Japan, Melanesia, Iran, Central and West Africa, the Caribbean and South America [2–4]. In Europe, North America and Australia, HTLV-1 infection is rare and mainly found in immigrants from endemic areas, and in their sexual partners [2,3]. However, the HTLV-1 prevalence has been poorly investigated in several areas of sub-Saharan Africa and in most parts of Asia [4,5]. HTLV-2 has a more restricted distribution than HTLV-1 and occurs primarily in the Americas and among pygmy tribes in Africa; Amerindians residing in North, Central, and South America show various rates of positivity for HTLV-2 (5 to 30%) [6,7].

Once acquired, HTLV-1 infections persist life-long, most patients remaining asymptomatic viral reservoirs ensuring the transmission chain, but about 4% develop adult T-cell leukemia/lymphoma (ATLL), a highly aggressive CD4+ T-cell malignancy. Type 1 virus has been associated with other diseases, ranging from a mild non-specific dermatitis and uveitis to a disabling HTLV-1-associated myelopathy/tropical spastic paraparesis (HAM/TSP) affecting 2–3% of the infected people [8]. HTLV-2 has been associated to hairy cell leukemia, erythrodermatitis and a growing number of neurologic disorders [9].

HTLV transmission is similar to that of human immunodeficiency virus (HIV), hepatitis C virus (HCV) and hepatitis B virus (HBV), but is less effective. The most important routes of HTLV-1/2 transmission are vertical transmission, unsafe sexual habits, transfusion of blood or blood products and needle sharing [10]. In particular, several factors have been associated with the sexual transmission of HTLV-1 infection, namely a presence of genital sores or ulcers, unprotected sexual intercourse, having multiple sexual partners, and a lifetime contact with an HTLV-1-infected partner [4,5].

Due to socio-economic and political crises in several countries in Africa, Eastern Europe and Central and Eastern Asia in recent decades, Italy has become a land of immigration from areas with an intermediate or high HTLV endemicity.

In literature there are few data about the seroprevalence of HTLV-1 and HTLV-2 infection in immigrants from endemic areas for this infection [11,12]; moreover, HTLV screening is currently not performed in blood donors in many European countries (Italy, Spain, Germany). Because of the poverty of data, we investigated for the presence of HTLV-1 and HTLV-2 infection a cohort of 1,498 immigrants from endemic areas (sub-Saharan Africa and Southern Asia) living in southern Italy (Naples, Caserta or Foggia) and consecutively observed at one of the five first-level clinical centers from January 2012 to July 2017.

## Materials and methods

### Ethics statement

All procedures used were in accordance with the international guidelines and with the Helsinki Declaration of 1975 and revised in 1983. The Ethics Committee of the Azienda Ospedaliera Universitaria of the University of Campania “Luigi Vanvitelli” approved the study (214/2012). All patients signed an informed consent for the collection and storage of biological samples and for the anonymous use of their data for research purposes.

### Methods

Estimating a prevalence about 3% [2–4] for HTLV infection with an accuracy estimate of 1%, the sample size had to be at least 1,118 subjects. Thus, to achieve the aim of the study, all 1,498 consecutive immigrants from HTLV-endemic areas (sub-Saharan Africa and Southern Asia), seeking care at one of the five first-level clinical centers between January 2012 and July 2017, were enrolled.

The first-level clinical centers involved in the present study are clinical centers of international charity organizations empowered by the Italian National Healthcare System, with proven experience in the clinical, psychological and legal management of vulnerable groups, as previously specified [13–15] Each first-level clinical center is an out-patients clinic of general medicine. They are located in Naples, Caserta and Foggia, an area that gives hospitality to a large population of refugees and undocumented immigrants from Africa, Central and Eastern Asia and Eastern Europe.

The study population consists of all the undocumented or refugee immigrants from sub-Saharan Africa and the India-Pakistan sub-continent consecutively seen for a clinical consultation. During the consultation, a physician from the clinical center and a cultural mediator explained to the immigrants the importance of testing for HBV, HCV, HIV and HTLV serum markers and offered them screening free of charge, in anonymity and in full accordance with the law on privacy.

Informed consent to participate in a surveillance and monitoring program covering different viral infections, including HTLV-1 and HTLV-2, was obtained on a voluntary basis. Of the interviewed immigrants 94.7% agreed to participate and were included in the present study. Patients who did not consent to participation in the study received the same treatment if they had any medical problem.

The participants were interviewed and relevant information was collected, including socio-demographic data and risk factors. More precisely, we recorded age, sex, geographical origin, time of immigration, level of education, religion, cohabitation details, sexual habits, a history of previous vaccination, surgery, dental care, tattooing, piercing, drug addiction, blood transfusion, tribal rituals (body scarification and/or infibulation) and abortion. In each case the clinical history was obtained with the help of a physician and a cultural mediator in the course of a prolonged, in-depth clinical consultation and counseling.

The serum samples obtained were transported to the laboratory of infectious diseases of the University of Campania Luigi Vanvitelli—Naples, where they were tested for HBsAg, anti-HBc, anti-HCV, anti-HIV and anti- HTLV-I/II. All undocumented immigrants and refugees received the results of their serological screening and full instructions on the prevention and transmission of the infections.

Serum samples were tested for HTLV by a commercial chemiluminescent microparticle immunoassay (Architect HTLV-I/II assay; Abbott, Wiesbaden-Delkenheim, Germany) and those positive by Western blot to confirm HTLV-1 or -2 infections (MP diagnostics HTLV- I/II blot 2.4). The Q6138 region was amplified by polymerase chain reaction (PCR) from whole blood sample [16] to confirm infection by the presence of proviral HTLV DNA in individuals who were positive for the Western blot test. Moreover, serum samples were tested for HBsAg, anti-HCV, anti-HIV and total anti-HBc by commercial immunoenzymatic assays (Abbott Laboratories, North Chicago, IL, USA: AxSYM HBsAg (V.2) M/S for HBsAg, AXSYM HCV 3.0 for anti-HCV, AXSYM HIV 1/2 COMBO for HIV, AXSYM core for total anti-HBc). Anti-HIV reactivity was always confirmed by a Western blot assay (Genelabs Diagnostics, Science Park Drive, Singapore), which identifies both HIV-1 and HIV-2 strains.

## Results

The demographic and serological data of the 1,498 immigrants obtained at the time of enrolment are shown in (Table 1.)

**Table 1:** Demographic, virological and clinical characteristics of all patients.

	Total
**N° of patients**	1,498
**Males, n° (%)**	1,334 (89.05)
**Age in years, median (range)**	26 14–72)
**Legal status, n° (%)**	
**Undocumented**	769 (51.33)
**Refugee**	729 (48.67)
**In Italy for months, median (range)**	5 (1–360)
**Religion, n° (%)**	
**Islamic**	988 (65.95)
**Christian**	487 (32.51)
**Buddhist**	6 (0.40)
**Not stated**	17 (1.13)
**Level of education in years, median (range)**	6 (0–22)
**Serological status, n° (%)**	
**HBsAg-positive**	153 (10.21)
**HBsAg-negative/anti-HBc positive**	605 (40.39)
**Anti-HCV-positive**	74 (4.94)
**Anti-HIV-positive**	24 (1.60)
**HBsAg-positive/anti-HIV-positive**	4 (0.27
**HBsAg-positive/anti-HCV-positive**	7 (0.47)
**Anti-HCV-positive/anti-HIV-positive**	2 (0.13)
**Risk factors, n° (%)**	
**Drug addiction**	25 (1.67)
**Unsafe sexual intercourse**	697 (46.53)
**Dental care**	227 (15.15)
**Surgery**	257 (17.15)
**Tattooing and piercing**	108 (7.21)
**Intramuscular injection**	852 (56.87)
**Tribal practices**	966 (64.49)
**Pregnant or breastfeeding women**	0

These subjects, prevalently young males and with a low level of education, had been living in Italy for a median period of 5 months; 48.26% of them were undocumented immigrants, 45.86% low income refugees and 5.87% did not state their legal status (Table 1). Most of them (79.7%) came from sub-Saharan Africa and 20.3% from southern Asia. More detailed information on the country of origin is shown in (Table 2).

**Table 2:** Region of origin of 1,498 immigrants enrolled.

	Total
**Sub-Saharan Africa, n° (%)**	1,194 (79.70)
**Benin**	9 (0.76)
**Burkina Faso**	42 (3.52)
**Cameroon**	16 (1.34)
**Cape Verde**	2 (0.17)
**Chad**	1 (0.08)
**Ivory Coast**	108 (9.04)
**Eritrea**	1 (0.08)
**Ethiopia**	2 (0.17)
**Gambia**	116 (9.71)
**Ghana**	167 (13.99)
**Guinea**	66 (5.53)
**Guinea Bissau**	16 (1.34)
**Liberia**	14 (1.17)
**Mali**	88 (7.37)
**Mauritania**	1 (0.08)
**Niger**	3 (0.25)
**Nigeria**	354 (29.65)
**Senegal**	158 (13.23)
**Sierra Leone**	7 (0.59)
**Somalia**	5 (0.42)
**Togo**	18 (1.51)
**Southern Asia, n° (%)**	304 (20.29)
**Afghanistan**	34 (11.18)
**Bangladesh**	135 (44.41)
**India**	3 (0.99)
**Pakistan**	109 (35.85)
**Sri Lanka**	23 (7.57)

(Table 1) shows the risk factors for parenteral and/or sexual acquisition of viral infections. Nearly 33% of immigrants stated invasive medical procedures such as surgery and dental care, 65% unsafe injective therapy, 8% tattooing and/or piercing, 57% tribal practices and nearly 47% unsafe sexual habits. Drug addiction was infrequently stated.

Of the 1,498 immigrants enrolled, only one (0.07%) was anti-HTLV-1 positive, 153 (10.2%) HBsAg-positive, 605 (40.39%) HBsAg-negative/anti-HBc-positive, 74 (4.9%) anti-HCV-positive, 24 (1.6%) anti-HIV-positive and 13 (0.87%) had a multiple infection (4 HBsAg/anti-HIV-positive, 2 anti-HCV/anti-HIV-positive and 7 HBsAg/anti-HCV-positive) (Table 1). No patient was anti-HTLV-2 positive. The immigrant found to be anti-HTLV-1-positive was a 27-year-old Nigerian Christian male with a low level of education (6 years) who had been living in Italy for 3 months and was asymptomatic and HTLV-1-RNA-negative. This subject was HBsAg, anti-HBc, anti-HCV and anti-HIV-1/2-negative and stated unsafe sexual habits and unsafe injection therapy.

## Discussion

In the present study, enrolling 1,498 African and Asian immigrants living in Italy, the prevalence of positive immunoezymatic tests for HTLV-1, confirmed by Western-blot test, was 0.07%. The only HTLV-1 subject infected was asymptomatic and HTLV-1-RNA-negative, in accordance with the literature data reporting a low prevalence of HTLV-1-RNA-positive patients among the anti-HTLV-1 positive [5,6].

In literature there are few data on the prevalence of HTLV-1/2 in immigrants [17] and, to our best knowledge, this is the first prospective study performed in Europe on HTLV infection in a population of undocumented and refugee immigrants from sub-Saharan Africa and the India-Pakistan subcontinent. In this study 1,194, subjects from several states of sub-Saharan Africa and 304 from the India-Pakistan area (India, Pakistan, Bangladesh, Sri-Lanka and Afghanistan) were screened for HTLV 1–2 antibodies. The subjects were enrolled at one of the five first-level medical centers with years of experience dealing with immigrants. Thus, the present study is, to our best knowledge, the first prospective study performed in Italy on an undocumented and refugee immigrant population providing valuable information on HTLV infections in relation to the demographics and geographical areas of origin. The subjects enrolled were young (median age 26 years), prevalently males and asymptomatic, and were representative of the immigrant population from developing countries living in Italy and Europe [13,14,18].

The data on the prevalence of HTLV 1–2 infection in this population are useful to devise proper healthcare strategies of screening for immigrant populations from different countries and to evaluate the need for screening of blood donors, today not indicated for this infection in Italy. In fact, in high-income countries, considering the low prevalence of HTLV-1 infection and the low rate of disease onset (below 10%), cost-effectiveness of universal HTLV-1 screening of blood donors is debated. However, the identification of HTLV-1 carriers is nevertheless important to avoid transmission through blood transfusion. The World Health Organization global database on blood safety does not include HTLV testing as one of its data collected from some countries around the world. The following countries test all blood donations for HTLV-1 and -2 antibody: Argentina, Australia, Brazil, USA, Canada, China (some regions)(13), Colombia, French West Indies, Iran, Israel, Jamaica, Japan, New Zealand, Saudi Arabia, Peru, Sweden, Taiwan, Uruguay and Venezuela. In Europe, HTLV-1/2 antibody testing is currently performed on all blood donations in France, Greece, Ireland, Netherlands, Portugal, Romania, United Kingdom and on first time blood donations in Denmark, Finland, Norway and Sweden [19].

Another important question is the high prevalence of HBV and HCV infection among immigrants living in Western countries [13,14,20,21], but fortunately in Italy screening for both of these viruses is planned at the time of blood donation.

Worthy of mention, in Italy the immigrant population constitutes one tenth of the whole population and its contribution to blood donation is strongly desired. Although HTLV-1 and HTLV-2 infections were rarely detected in the immigrant population investigated in this study, we believe that every effort must be made to make all blood donations as safe as possible. It may be advisable for the Italian Healthcare Authorities to start screening for HTLV-1 and HTLV-2 infections all donors from endemic countries at least on their first donation, a practice useful for a correct cost-effectiveness analysis and for a conclusive decision on this topic. In fact, even if the prevalence of HTLV-1 and HTLV-2 is low, it is not negligible and, considering the transmissibility through blood and sexual route, could represent a significant problem in Western countries both in terms economic and public health. However, a cost-effectiveness study is needed to clarify this point.

## Supporting information

S1 Checklist STROBE checklist. (DOCX)
