# Supplementary material for: Low prevalence of HTLV1/2 infection in a population of immigrants living in southern Italy
Source: PLoS Negl Trop Dis. 2018 Jun 25;12(6):e0006601. doi: 10.1371/journal.pntd.0006601 (PMC6034900; doi:10.1371/journal.pntd.0006601)
Supplement: S1 Checklist — (DOCX) [file pntd.0006601.s001.docx]

STROBE Statement—checklist of items that should be included in reports of observational studies

|  | Item No. | Recommendation | Page  No. | Relevant text from manuscript |
| --- | --- | --- | --- | --- |
| **Title and abstract** | 1 | (*a*) Indicate the study’s design with a commonly used term in the title or the abstract | Abstract  Methods, first paragraph | We performed a cross-sectional study including all 1,498 consecutive immigrants |
|  |  | (*b*) Provide in the abstract an informative and balanced summary of what was done and what was found | Abstract | Aims. To assess the prevalence and clinical presentation of HTLV-1 and HTLV-2 infections in a cohort of immigrants living in southern Italy.  Methodology. We performed a cross-sectional study screening for antibody to HTLV-1/2 infection in 1,498 consecutive immigrants born in endemic areas (sub-Saharan Africa or southern-Asia) by a commercial chemiluminescent microparticle immunoassay. If confirmed in a Western blot assay, which differentiates anti-HTLV-1 from anti-HTLV-2, the positive sera were tested for specific HTLV RNA by a home-made PCR.  Results. The immigrants investigated were more frequently males (89.05%), young (median age 26 years), with a low level of education (median schooling 6 years), born in sub-Saharan Africa (79.70%). They had been living in Italy for a median period of 5 months. Only one (0.07%) subject was anti-HTLV-1 -positive/HTLV-1 RNA-negative; he was an asymptomatic 27-year-old male from Nigeria with 6 years’ schooling who stated unsafe sexual habits and unsafe injection therapy.  Conclusions. The data suggest screening for HTLV1 and HTLV-2 infections all blood donors  To Italy from endemic countries at least on their first donation, a practice useful for a correct cost-effectiveness analysis and a conclusive decision on this topic. |
| Introduction | | | |  |
| Background/rationale | 2 | Explain the scientific background and rationale for the investigation being reported | Introduction, last paragraph | Human T cell leukemia virus type 1 (HTLV-1) or 2 (HTLV-2) infection has a worldwide distribution, with an estimate of up to 15–20 million people affected [1]. The prevalence changes substantially according to the geographical area, and is higher in specific risk groups such as intravenous drug users and sex workers [2]. In Europe, North America and Australia, HTLV-1 infection is rare and mainly found in immigrants from endemic areas, and in their sexual partners [3]. HTLV transmission is similar to that of human immunodeficiency virus (HIV), hepatitis C virus (HCV) and hepatitis B virus (HBV), but is less effective. Due to socio-economic and political crises in several countries in Africa, Eastern Europe and Central and Eastern Asia in recent decades, Italy has become a land of immigration from areas with an intermediate or high HTLV endemicity. |
| Objectives | 3 | State specific objectives, including any prespecified hypotheses | Introduction, last paragraph | We investigated for the presence of HTLV-1 and HTLV-2 infection in a cohort of 1,498 immigrants from endemic areas (sub-Saharan Africa and Southern Asia) living in southern Italy (Naples, Caserta or Foggia) and consecutively observed at one of the five first-level clinical centers from January 2012 to July 2017. |
| Methods | | | |  |
| Study design | 4 | Present key elements of study design early in the paper | Methods, second paragraph | We performed a cross-sectional study including all 1,498 consecutive immigrants from HTLV-endemic areas (sub-Saharan Africa and Southern Asia) seeking care at one of the five first-level clinical centers between January 2012 and July 2017 were enrolled |
| Setting | 5 | Describe the setting, locations, and relevant dates, including periods of recruitment, exposure, follow-up, and data collection | Methods, first three paragraphs | All rilevant |
| Participants | 6 | (*a*) *Cohort study*—Give the eligibility criteria, and the sources and methods of selection of participants. Describe methods of follow-up  *Case-control study*—Give the eligibility criteria, and the sources and methods of case ascertainment and control selection. Give the rationale for the choice of cases and controls  *Cross-sectional study*—Give the eligibility criteria, and the sources and methods of selection of participants | Not applicable  Not applicable  Methods, second paragraph | immigrants from HTLV-endemic areas (sub-Saharan Africa and Southern Asia) |
|  |  | (*b*) *Cohort study*—For matched studies, give matching criteria and number of exposed and unexposed  *Case-control study*—For matched studies, give matching criteria and the number of controls per case | Not applicable  Not applicable |  |
| Variables | 7 | Clearly define all outcomes, exposures, predictors, potential confounders, and effect modifiers. Give diagnostic criteria, if applicable | Not applicable |  |
| Data sources/ measurement | 8* | For each variable of interest, give sources of data and details of methods of assessment (measurement). Describe comparability of assessment methods if there is more than one group | Not applicable |  |
| Bias | 9 | Describe any efforts to address potential sources of bias | Not applicable |  |
| Study size | 10 | Explain how the study size was arrived at | Methods, first paragraph | We included all subjects which fell within the inclusion criteria |

Continued on next page

| Quantitative variables | 11 | Explain how quantitative variables were handled in the analyses. If applicable, describe which groupings were chosen and why | Not applicable |  |
| --- | --- | --- | --- | --- |
| Statistical methods | 12 | (*a*) Describe all statistical methods, including those used to control for confounding | Not applicable |  |
|  |  | (*b*) Describe any methods used to examine subgroups and interactions | Not applicable |  |
|  |  | (*c*) Explain how missing data were addressed | Not applicable |  |
|  |  | (*d*) *Cohort study*—If applicable, explain how loss to follow-up was addressed  *Case-control study*—If applicable, explain how matching of cases and controls was addressed  *Cross-sectional study*—If applicable, describe analytical methods taking account of sampling strategy | Not applicable  Not applicable  Not applicable |  |
|  |  | (*e*) Describe any sensitivity analyses | Not applicable |  |
| Results | | | | |
| Participants | 13* | (a) Report numbers of individuals at each stage of study—eg numbers potentially eligible, examined for eligibility, confirmed eligible, included in the study, completing follow-up, and analysed | not applicable |  |
|  |  | (b) Give reasons for non-participation at each stage not applicable |  |  |
|  |  | (c) Consider use of a flow diagram not applicable |  |  |
| Descriptive data | 14* | (a) Give characteristics of study participants (eg demographic, clinical, social) and information on exposures and potential confounders | Results, first paragraph | All relevant |
|  |  | (b) Indicate number of participants with missing data for each variable of interest | Not applicaple |  |
|  |  | (c) *Cohort study*—Summarise follow-up time (eg, average and total amount) | Not applicaple |  |
| Outcome data | 15* | *Cohort study*—Report numbers of outcome events or summary measures over time | Not applicaple |  |
|  |  | *Case-control study—*Report numbers in each exposure category, or summary measures of exposure | Not applicaple |  |
|  |  | *Cross-sectional study—*Report numbers of outcome events or summary measures | Not applicaple |  |
| Main results | 16 | (*a*) Give unadjusted estimates and, if applicable, confounder-adjusted estimates and their precision (eg, 95% confidence interval). Make clear which confounders were adjusted for and why they were included | Not applicaple |  |
|  |  | (*b*) Report category boundaries when continuous variables were categorized | Not applicaple |  |
|  |  | (*c*) If relevant, consider translating estimates of relative risk into absolute risk for a meaningful time period | Not applicaple |  |

Continued on next page

| Other analyses | 17 | Report other analyses done—eg analyses of subgroups and interactions, and sensitivity analyses | Not applicaple |  |
| --- | --- | --- | --- | --- |
| Discussion | | | | |
| Key results | 18 | Summarise key results with reference to study objectives | Conclusion, first paragraph | All relevant |
| Limitations | 19 | Discuss limitations of the study, taking into account sources of potential bias or imprecision. Discuss both direction and magnitude of any potential bias | Conclusion, second paragraph | All relevant |
| Interpretation | 20 | Give a cautious overall interpretation of results considering objectives, limitations, multiplicity of analyses, results from similar studies, and other relevant evidence | Conclusion, third paragraph | All relevant |
| Generalisability | 21 | Discuss the generalisability (external validity) of the study results | Not applicable |  |
| Other information | |  | | |
| Funding | 22 | Give the source of funding and the role of the funders for the present study and, if applicable, for the original study on which the present article is based |  | This study was supported in part by a grant from Gilead Sciences S.r.l. ‘L’infezione da HBV nelle popolazioni speciali (donne in gravidanza, popolazioni immigrate, popolazioni in età pediatrica): progetti di awareness ed accesso alla diagnosi’ Fellowship Program 2011, 2013 and 2016; and by a grant from the 2014 goSHAPE program. The funders did not have a role for the present study and |

*Give information separately for cases and controls in case-control studies and, if applicable, for exposed and unexposed groups in cohort and cross-sectional studies.

**Note:** An Explanation and Elaboration article discusses each checklist item and gives methodological background and published examples of transparent reporting. The STROBE checklist is best used in conjunction with this article (freely available on the Web sites of PLoS Medicine at http://www.plosmedicine.org/, Annals of Internal Medicine at http://www.annals.org/, and Epidemiology at http://www.epidem.com/). Information on the STROBE Initiative is available at www.strobe-statement.org.
